# Regulation of *Laminaria* Polysaccharides with Different Degrees of Sulfation during the Growth of Calcium Oxalate Crystals and their Protective Effects on Renal Epithelial Cells

**DOI:** 10.1155/2021/5555796

**Published:** 2021-08-26

**Authors:** Wei-Bo Huang, Guo-Jun Zou, Gu-Hua Tang, Xin-Yuan Sun, Jian-Ming Ouyang

**Affiliations:** ^1^Institute of Biomineralization and Lithiasis Research, Jinan University, Guangzhou 510632, China; ^2^Department of Urology, Guangzhou Institute of Urology, Guangdong Key Laboratory of Urology, The First Affiliated Hospital of Guangzhou Medical University, Guangzhou Medical University, Guangzhou, Guangdong 510230, China

## Abstract

The original *Laminaria* polysaccharide (LP0) was sulfated using the sulfur trioxide-pyridine method, and four sulfated *Laminaria* polysaccharides (SLPs) were obtained, namely, SLP1, SLP2, SLP3, and SLP4. The sulfated (–OSO_3_^–^) contents were 8.58%, 15.1%, 22.8%, and 31.3%, respectively. The structures of the polysaccharides were characterized using a Fourier transform infrared (FT-IR) spectrometer and nuclear magnetic resonance (NMR) techniques. SLPs showed better antioxidant activity than LP0, increased the concentration of soluble Ca^2+^ in the solution, reduced the amount of CaOx precipitation and degree of CaOx crystal aggregation, induced COD crystal formation, and protected HK-2 cells from damage caused by nanometer calcium oxalate crystals. These effects can inhibit the formation of CaOx kidney stones. The biological activity of the polysaccharides increased with the content of –OSO_3_^−^, that is, the biological activities of the polysaccharides had the following order: LP0 < SLP1 < SLP2 < SLP3 < SLP4. These results reveal that SLPs with high –OSO_3_^−^ contents are potential drugs for effectively inhibiting the formation of CaOx stones.

## 1. Introduction

The prevalence and incidence of kidney stones are on the rise globally, and no effective drug to treat or prevent the disease is currently available [1]. Calcium oxalate (CaOx) is the main inorganic component of kidney stones, accounting for about 70%–80% [2]. CaOx in kidney stones mainly exists in the form of calcium oxalate monohydrate (COM) and calcium oxalate dihydrate (COD) [3]. COM is the most stable phase of thermodynamics and the most common form of stones. The incidence of stones caused by COM is twice that caused by COD [4]. Compared with COD, COM has a greater affinity for renal tubular cells [5] and is more difficult to be excreted in the urine. Therefore, COM in the body is more likely to induce kidney stone formation than COD crystals.

Oxidative stress-induced damage to renal tubular epithelial cells and decrease in inhibitor molecules in urine or in their activities are the important reasons for the formation of CaOx stones [6, 7]. Increasing the concentrations and/or activities of inhibitors in the urine, removing excess free radicals in the body, and protecting renal tubular epithelial cells from oxidative damage may inhibit the formation and recurrence of kidney stones.

The chemical structures of plant polysaccharides are very similar to the structure of CaOx crystal growth inhibitor glucosamine (GAGs), which is commonly found in urine. Plant polysaccharides contain a large number of anionic groups, such as sulfate groups (–OSO_3_^–^) and carboxyl groups (–COO^–^), and can thus be used to inhibit the formation of CaOx kidney stones. Huang et al. [8] showed that plant polysaccharides can inhibit the growth and aggregation of COM and induce the formation of COD. Gangu et al. [9] found that Gum Arabic (GA) can regulate the phase and morphology of CaOx, promote the formation of COD crystals, and inhibit the growth of COM crystals.

High concentrations of oxalic acid and CaOx or CaP crystals activate NADPH oxidase, produce excessive reactive oxygen species (ROS), and induce the inflammation and oxidative damage of renal epithelial cells, leading to the death of renal epithelial cells [10]. Tubular epithelial cell injury can induce the high expression of cell surface adhesion molecules, such as hyaluronic acid, osteopontin (OPN), and CD44, resulting in the accumulation of CaOx crystals in the kidney [11, 12]. Xi et al. [13] showed that high concentrations of calcium stimulate the attachment of CaOx crystals to rat renal tubular epithelial cells by inducing OPN expression, leading to the deposition of crystals in the kidney and the formation of CaOx stones. Plant polysaccharides can protect renal epithelial cells from oxidative damage by inhibiting the adhesion of CaOx crystals to cells. For example, Zhao et al. [14] found that tea polysaccharides can effectively protect HK-2 cells from COM damage, thus increasing cell viability, restoring cell morphology, increasing lysosome integrity, and reducing crystal adhesion. These features inhibit the production of kidney stones.

Plant polysaccharides not only effectively remove superoxide and various free radicals, such as hydroxyl, DPPH, and ABTS, but also reduce the level of lipid peroxidation, are less toxic, and have a lower number of side effects on the body. The biological activity of plant polysaccharides is not only related to its monosaccharide composition and molecular weight but also closely related to the content of active groups (such as –OSO_3_^−^ and –COOH) in polysaccharide molecules. These active groups reduce the rate of negative charge loss on cell surfaces and repair the charge barrier. Chen et al. [15] obtained three types of sulfated polysaccharides with a sulfated contents of 9.11%, 10.33%, and 21.44% from *Ganoderma atrum*. The scavenging ability of these components on DPPH free radicals is positively correlated with their –OSO_3_^–^ contents, that is, polysaccharides with high –OSO_3_^−^ contents are more resistant to oxidation. Chen et al. [16] modified *Momordica charantia* polysaccharide (MCP) by sulfation to obtain sulfated *Momordica charantia* polysaccharide (S-MCP) with a substitution degree of 0.45. S-MCP has a greater scavenging ability for superoxide anions than MCP. Lu et al. [17] modified chitooligosaccharides (COS) through sulfation to prepare COS-SI and COS-SII with substitution degrees of 0.8 and 1.9, respectively. Their protective effects on H_2_O_2_-induced MIN6 cell damage had the order COS-SII > COS-SI > COS, indicating that increasing the substitution degree of –OSO_3_^−^ in polysaccharides prevent H_2_O_2_-induced oxidative damage in MIN6 cells.

*Laminaria* is an important economic seaweed and is widely found in the seas of China, Japan, and Korea. It is also the favorite food of some Asians. In the past thousand years, the Chinese has used it as a traditional medicine to treat edema disease [18]. *Laminaria* polysaccharide (LP) is the main active component of *Laminaria*. The antioxidant activity of LP was positively correlated with the –OSO_3_^–^ content of polysaccharide. For example, Wang et al. [19] obtained F1, F2, and F3 components with –OSO_3_^−^ contents of 23.3%, 36.41%, and 36.67% from *Laminaria*. The scavenging ability of the components on superoxide radicals had the following order: F3 > F2 > F1, which indicates that the strength of the antioxidant activity of the polysaccharides increases with –OSO_3_^–^ content. Wang et al. [20] extracted fucoidan (FPS) with a –OSO_3_^–^ content of 27.56% from *Laminaria* and sulfated FPS to obtain SO_3_-FPS with a –OSO_3_^–^ content of 36.63%. The scavenging ability of SO_3_-FPS on hydroxyl radicals was significantly stronger than that of FPS.

However, the –OSO_3_^–^ content of polysaccharide has a great relationship with its producing area and extraction method [21–23]. For example, Yoon et al. [21] extracted three polysaccharide components (S1, S2, and S3) from *Laminaria cichorioides* in the East Sea of Korea, and the –OSO_3_^–^ content in S1, S2, and S3 were 0.09%, 2.19%, and 1.38%, respectively. Cui et al. [22] extracted *Laminaria japonica* polysaccharide (LJPA-P) from *Laminaria japonica* collected from Qingdao, Shandong, China, by a joint extraction of hot water and citric acid, and the –OSO_3_^–^ content of LJPA-P was 4%. However, the polysaccharides isolated and purified by LJPA-P (LJPA-P1 and LJPA-P2) could not even detect the –OSO_3_^–^ content. Saha et al. [23] collected crude *Laminaria angustata* polysaccharide (WEP) from the Okha coast of Gujarat, India. The –OSO_3_^–^ content of WEP was 3% and purified *Laminaria angustata* polysaccharide (F2) with a –OSO_3_^–^ content of 4.2%. These polysaccharides with low –OSO_3_^–^ content need to be sulfated to enhance their biological activity.

In this study, LP0 was sulfated to different degrees, and four kinds of SLPs (SLP1–SLP4) with –OSO_3_^−^ contents of 8.58%, 15.1%, 22.8%, and 31.3% were obtained. The antioxidant activities, the ability to regulate CaOx crystals growth, and protective effects on renal epithelial cells of these SLPs were studied. The SLPs offer development prospects for drugs used in preventing and treating CaOx kidney stones.

## 2. Materials and Methods

### 2.1. Reagents and Apparatus

*Reagents.* Deuterium oxide (D_2_O, 99.9%, sigma) was purchased from Macklin. Dimethyl sulfoxide (AR, >99%), formamide (ACS grade, ≥99.5%), potassium bromide (KBr, AR, 99.0%), and sulfur trioxide-pyridine complex (97%) were all purchased from Aladdin. Hydrogen peroxide 30% (H_2_O_2_), sodium oxalate (Na_2_Ox), and calcium chloride dihydrate (CaCl_2_•2H_2_O) were all purchased from Guangzhou Chemical Reagent Factory of China (Guangzhou, China). The experimental water is double-distilled water.

DMEM/F-12 culture medium and fetal bovine serum were purchased from Gibco company. Cell counting kit (CCK-8) was purchased from Dojindo Laboratory (Kumamoto, Japan). 2′,7′-dichlorodihydrofluorescein diacetate (DCFH-DA) was purchased from Shanghai Beyotime Bio-Tech Co., Ltd. (Shanghai, China). Human kidney proximal tubular epithelial (HK-2) cells were purchased from Shanghai Cell bank of Chinese Academy of Sciences.

*Apparatus.* Nuclear Magnetic Resonance Analyzer (Varian Bruker-600 MHz, Germany); Multifunctional Microplate Reader (SafireZ, Tecan, Switzerland); Fourier Transform Infrared Absorption Spectrometer (EQUINOX55, Bruker, Germany); Constant temperature water bath (Ningbo Saifu Experimental Instrument Factory); D/max2400 X-ray powder diffractometer (Rigaku, Japan); X-L type environmental scanning electron microscope (ESEM, Philips, Eindhoven, Netherlands); OPTIMA-2000DV inductively coupled plasma emission spectrometer (ICP-AES, USA PE company); Nanoparticle sizer (Nano-ZS, Malvem, UK); Inverted fluorescence microscope (OLYMPUS, U-HGLGPS, Japan); Optical microscope (OLYMPUS, TH4-200, Japan); Gas chromatography-mass spectrometer (Agilent, USA).

### 2.2. Preparation of *Laminaria* Polysaccharide (LP0)

Samples of *Laminaria* were collected from the Guangdong of China from August to September 2019. The material was sorted, washed, and dried immediately by forced air circulation at 50–60°C. A hot water-extracted polysaccharide was obtained from the algal powder of Laminaria (diameter, 100-200 *μ*m) with 90-fold volumes of distilled water for 5 h at 90°C. After centrifugation to remove residues (7000 rpm, 10 min), the supernatant was concentrated to one-third of the volume in a vacuum rotary evaporator. The concentrated solution was then precipitated with 3 volumes of the absolute ethanol overnight at 4°C. The precipitates were collected by centrifugation (3500 g, 10 min) and, then, resolved in warm water. Proteins were removed using the Sevag method. The supernatant of polysaccharides was dialyzed (Mw of cutoff 500 Da, Regenerated Cellulose) in distilled water for 72 h and vacuum freeze-dried.

### 2.3. Measurement of LP0 Average Molecular Weight and Monosaccharide Composition

#### 2.3.1. LP0 Average Molecular Weight

The molecular weight of LP0 was determined by the Ubbelohde viscosity method at 25 ± 0.2°C. After measuring the fall time of polysaccharide solution in the viscometer, specific (*η*_sp_) and relative (*η*_*r*_) viscosity was calculated according to the formulas *η*_r_ = *T*_i_/*T*_0_ and *η*_sp_ = *η*_r_ − 1, where *T*_*i*_ and *T*_0_ were the falling time of LP0 solution and deionized water, respectively. Using the one-point method formula, the intrinsic viscosity [*η*] = [2(*η*_sp_ − ln*η*_r_)]^1/2^/*c* is obtained, where *c* is the concentration of the polysaccharide solution to be tested. The molecular weight of LP0 (*M*) was calculated through its [*η*] value. Because the relationship between the intrinsic viscosity [*η*] of the polymer solution and its molecular weight *M* can be expressed by the Mark-Houwink empirical equation, that is: [*η*] = *κM*^*α*^, where *κ* and *α* are the two parameters of the empirical equation. For LP0: *κ* = 8.21 × 10^−3^, *α* = 0.782. Take the average of 3 parallel experiments.

#### 2.3.2. LP0 Monosaccharide Composition by GC-MS

The monosaccharide component of LP0 was detected by GC-MS according to our previous study [24]. The details are as follows: 10 mg of LP0 polysaccharide was added to a 121°C sealed container containing 2.5 mol/L trifluoroacetic acid (TFA, 2 mL) for 90 min. The solution was concentrated to dryness under reduced pressure; then, the TFA was removed with MeOH to a neutral solution and concentrated to dryness under reduced pressure. The residue was dissolved in 2 mol/L NH_4_OH (1 mL) and 1 mol/L fresh NaBD_4_ (1 mL). The reaction was carried out at room temperature for 2.5 h and stirring at room temperature. Then, two drops of acetic acid were added to decompose excess NaBD_4_ until no bubbles producing. The solution was concentrated to dryness under reduced pressure. The filtrate was added MeOH to remove boric acid and dried in vacuo. Add 1 mL acetic anhydride and acetylate at 100°C for 2.5 h.

The acetylated product was extracted with dichloromethane. The organic layer was washed with distilled water, dried, and analyzed by GC-MS. The HP-5MS capillary column (15 m × 250 *μ*m × 0.25 *μ*m) was programmed and the temperature was raised from 135°C to 180°C at 0.5°C/min, then to 190°C at 10°C/min and up to 310°C at 40°C/min. Helium acts as carrier gas, column flow rate 0.6 mL/min. The acetylated product was identified by debris ions in GC-MS and relative retention times in GC. The structure is identified by peaks and assessed by peak area. Standard monosaccharides (rhamnose, arabinose, fucose, sucrose, maltose, glucose, raffinose, fructose, and galactose) are used as references.

### 2.4. Sulfation and Characterization of LP0

#### 2.4.1. Sulfation of LP0

According to the reference [25, 26], the specific steps are as follows: accurately weigh 500 mg of LP0 into a 250 mL Erlenmeyer flask, add 30 mL of dimethyl sulfoxide, and stir at room temperature for 1 hour to completely dissolve the polysaccharide. Refer to Table 1 to add sulfur trioxide pyridine complex and 5 mL of formamide (sulfur trioxide-pyridine complex was dissolved in formamide in advance to make it transparent), and reacted for a certain period of time (0.5, 2, and 8 h) in a water bath. After the reaction, the sample was cooled to room temperature with ice water, neutralized to pH = 7.0 with 2.0 mol/L NaOH solution, and then transferred to a dialysis bag (Mw = 500 Da) for dialysis for 4 days, until the solution in the dialysis bag changed from light yellow to transparent. After dialysis, it is evaporated and concentrated to 3 mL at 50°C. The polysaccharide is precipitated with absolute ethanol. After standing overnight, it is centrifuged and dried to obtain sulfated polysaccharides SLP1, SLP2, SLP3, and SLP4 containing different -OSO_3_^−^ content.

#### 2.4.2. Detection of -OSO_3_^−^ Content in Polysaccharides

The BaCl_2_-gelatin turbidimetric method was used to determine the -OSO_3_^−^ content of each SLPs [27], and K_2_SO_4_ was used as a standard to draw a standard working curve. The regression equation was as follows: *Y* = 4.72803*X* + 0.01723, *R*^2^ = 0.99498, *n* = 11.

#### 2.4.3. FT-IR Spectrum of SLPs

Take 2.0 mg dried polysaccharide sample and 200 mg KBr, grind it to powder with an agate mortar, press the tablet, and scan in the wave number range of 4000-400 cm^−1^.

#### 2.4.4. ^1^H NMR and ^13^C NMR Spectra of SLPs

According to the reference [28], weigh 20 mg dried polysaccharide sample, dissolve it in a nuclear magnetic tube containing 0.55 mL of deuterium oxide (D_2_O). After being completely dissolved, it was placed in the magnetic field of a nuclear magnetic resonance spectrometer for detection.

### 2.5. The Regulation Effect of Different SLPs on the Growth of CaOx Crystals

1. *CaOx Crystal Synthesis.* Add 20 mL CaCl_2_ solution with a concentration of 22 mmol/L to a set of beakers, then add a certain amount of SLPs with different sulfation degrees, and add distilled water to make up to 24 mL. It is placed in a 37°C constant temperature water bath and magnetically stir for 5 minutes. Add 20 mL of 22 mmol/L Na_2_Ox solution to make the final volume of the system 44 mL. At this time, c(Ca^2+^) = c(Ox^2−^) = 10 mmol/L in the solution, and the final polysaccharide concentration is 0.4 g/L, 0.8 g/L, 1.2 g/L, 1.6 g/L, and 2.4 g/L. The solution was stirred in a constant temperature water bath for 10 min, then transferred to a constant temperature water bath at 37°C and allowed to stand for 2 h, and centrifuged. The bottom CaOx precipitate obtained after centrifugation is dried in an oven. The soluble Ca^2+^ ions concentration in the supernatant of the solution is measured by the ICP method. The bottom CaOx precipitate is dried in a dryer and weighed. After weighing, XRD, FT-IR, Zeta potential, and SEM tests are performed
2. *XRD Detection.* Take a certain amount of crystals and grind them into powder in a mortar for detection. The relative percentages of COM and COD in CaOx are calculated by the *K* value method according to the XRD spectrum

(1) $$\begin{array}{c} \text{COD}\%=\frac{I_{\text{COD}}}{I_{\text{COM}}+I_{\text{COD}}}\times 100. \end{array}$$

In the formula, *I*_COM_ and *I*_COD_ are the intensities of the main diffraction peak ($\bar{1}01$) crystal plane (*d* = 0.593 nm) of COM and the main diffraction peak (200) crystal plane (*d* = 0.618 nm) of COD, respectively.

(3) *FT-IR Detection.* Take 2.0 mg sample and 200 mg KBr mixed, grind it to powder in an agate mortar, press the tablet, and scan in the wave number range of 4000~400 cm^−1^
(4) *Observation by SEM.* Weigh 1.0 mg sample, ultrasonically disperse it in 5 mL absolute ethanol at low power, spot the sample on a 10 mm × 10 mm glass slide, and dry at room temperature. After spraying gold on the sample, observe the crystal morphology under SEM
(5) *Zeta Potential Measurement.* Weigh 1.0 mg sample, ultrasonically disperse it in 3 mL pure water, and measure with a nanoparticle sizer under a constant temperature of 25°C

### 2.6. Antioxidant Activity of Different SLPs

The hydroxyl radical (•OH) scavenging and DPPH free radical scavenging capabilities of polysaccharides in vitro were detected by the H_2_O_2_/Fe^2+^ system method and DPPH reagent method, respectively. Parallel three times and take the average.

### 2.7. Cell Experiment

#### 2.7.1. Cell Viability Detection

Inoculate the cell suspension (density 1.0 × 10^5^ cells/mL) in a 96-well plate, 100 *μ*L per well, and incubate for 24 h in an incubator at 37°C, 5% CO_2_, and saturated humidity to make the cells converge into a monolayer. The cells were divided into four groups: (a) cell-free culture medium group; (b) normal control group: only serum-free medium was added; (c) crystal damage group: 200 *μ*g/mL COD crystals prepared with serum-free medium was added; (d) polysaccharide protection group: 20, 40, and 80 *μ*g/mL *Laminaria* polysaccharide solution with -OSO_3_^−^ content of 0.73% (LP0), 8.58% (SLP1), 15.1% (SLP2), 22.8% (SLP3), and 31.3% (SLP4) was added to react with 200 *μ*g/mL COD for 15 min; then, it was added to normal cells and incubated for 12 h. After reaching the action time, add 10 *μ*L of CCK-8 reagent to each well, incubate for 2 h at 37°C, measure the absorbance (A) at 450 nm with a microplate reader, and measure the cells under each condition in parallel in 5 replicate wells. Calculate the average value of A to test the protective ability of polysaccharides.

The concentration of polysaccharide in cell experiment was controlled by the following methods: serum-free medium was used to prepare a polysaccharide solution with a higher concentration (500 *μ*g/mL), and then, a certain volume of the above polysaccharide solution is taken according to the target concentration (such as 20, 40, or 80 *μ*g/mL) of the experiment, combined with the amount of other reagents added in the experiment, and finally diluted to the same volume with serum-free medium.

#### 2.7.2. Cytotoxicity Detection of Polysaccharides

Inoculate the cell suspension (density 1.0 × 10^5^ cells/mL) in a 96-well plate, 100 *μ*L per well, and incubate for 24 h in an incubator at 37°C, 5% CO_2_, and saturated humidity to make the cells converge into a monolayer. The cells were divided into two groups: (a) normal control group: only serum-free medium was added; (b) polysaccharide group: 20, 40, and 80 *μ*g/mL *Laminaria* polysaccharide solution were added to normal cells for 24 h. After 24 h of incubation, add 10 *μ*L of CCK-8 reagent to each well, incubate at 37°C for 2 h, measure the absorbance (A) at 450 nm with a microplate reader, and measure the cells under each condition in parallel in 5 replicate wells. The average value of A is used to detect the toxicity of polysaccharides.

#### 2.7.3. Detection of Reactive Oxygen Species (ROS) Levels

Inoculate the cell suspension with a concentration of 1.0 × 10^5^ cells/mL in a 6-well culture plate with a volume of 2 mL/well. After incubating for 24 h, aspirate the culture solution and wash the cells twice with PBS. The experimental model is divided into three groups: (a) normal control group: only serum-free medium was added; (b) crystal damage group: 200 *μ*g/mL COD crystals prepared with serum-free medium was added; (c) polysaccharide protection group: 80 *μ*g/mL *Laminaria* polysaccharide solution with -OSO_3_^−^ content of 0.73% (LP0), 8.58% (SLP1), 15.1% (SLP2), 22.8% (SLP3), and 31.3% (SLP4) was added to react with 200 *μ*g/mL COD for 15 min; then, it was added to normal cells and incubated for 12 h. After reaching the action time, aspirate the supernatant in the 6-well plate, add 500 *μ*L DCFH-DA dilution to each well, incubate in a 37°C incubator for 30 min, wash the cells three times with PBS, and observe under a fluorescence microscope. The ImageJ software was used for semiquantitative analysis of ROS fluorescence.

#### 2.7.4. Cell Morphology Observation

Inoculate the cell suspension with a concentration of 1.0 × 10^5^ cells/mL in a 6-well culture plate with a volume of 2 mL/well. After incubating for 24 h, aspirate the culture solution and wash the cells twice with PBS. The experimental model is divided into three groups: (a) normal control group: only serum-free medium was added; (b) crystal damage group: 200 *μ*g/mL COD crystals prepared with serum-free medium was added; (c) polysaccharide protection group: 80 *μ*g/mL *Laminaria* polysaccharide solution with -OSO_3_^−^ content of 0.73% (LP0), 8.58% (SLP1), 15.1% (SLP2), 22.8% (SLP3), and 31.3% (SLP4) was added to react with 200 *μ*g/mL COD for 15 min; then, it was added to normal cells and incubated for 12 h. After reaching the action time, observe the cell morphology under a microscope.

### 2.8. Statistical Analysis

Experimental data were expressed by mean ± standard deviation ($\bar{x}\pm \text{SD}$). The experimental results were statistically analyzed by the IBM SPSS Statistics 26 software, and the differences between the means of each experimental group and the control group were analyzed by Tukey. *P* < 0.05 indicates a significant difference; *P* < 0.01 indicates a very significant difference. *P* > 0.05 indicates no significant difference.

## 3. Results

### 3.1. Measurement of LP0 Average Molecular Weight and Monosaccharide Composition

**Figure 1:**
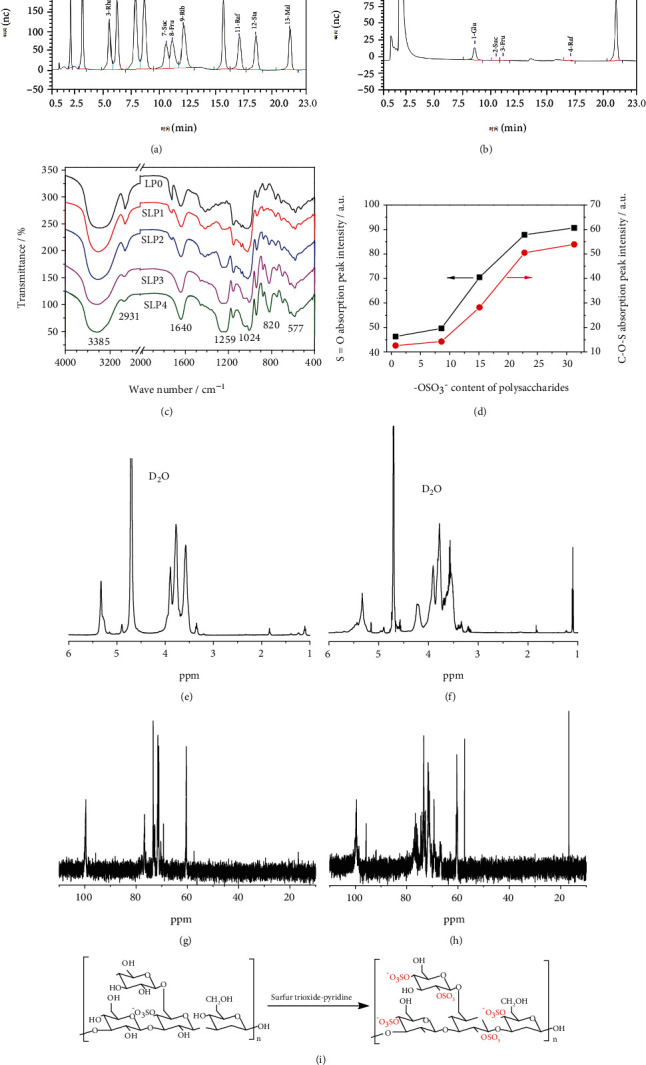
Characterization of SLPs with different -OSO_3_^−^ content. (a, b) GC-MS analysis. (c) FT-IR spectrum. (d) The absorption peak intensity of S=O asymmetric stretching vibration and C-O-S stretching vibration varies with the -OSO_3_^−^ content of polysaccharides. (e) ^1^H NMR spectrum of LP0. (f) ^1^H NMR spectrum of SLP2. (g) ^13^C NMR spectrum of LP0. (h) ^13^C NMR spectrum of SLP2. (i) LP0 sulfation reaction equation.

The average molecular weight of LP0 is determined to be 1788 ± 78 Da by the Ubbelohde viscosity method. Figures 1(a) and 1(b) present the GC-MS results of standard monosaccharides and LP0. The majority of LP0 is composed of maltose and glucose with traces of sucrose, fructose, and raffinose. The mass ratio of maltose to glucose to raffinose to fructose to sucrose is 12.49 : 1.42 : 0.09 : 0.08 : 0.03.

### 3.2. Sulfation and Characterization of LP0

#### 3.2.1. Sulfation of LP0

LP0 was sulfated using the sulfur trioxide-pyridine method [25, 26]. By controlling reaction time, the concentration of the reactant (sulfur trioxide pyridine) and temperature, four SLPs, namely, SLP1, SLP2, SLP3, and SLP4 with sulfate groups (–OSO_3_^–^) content of 8.58%, 15.1%, 22.8%, and 31.3%, respectively, were obtained (Table 1).

As shown in Table 1, the –OSO_3_^–^ contents of the polysaccharides increase with reaction time, reactant concentration, and reaction temperature. However, SLP4 with an –OSO_3_^–^ content of as high as 31.3% can be prepared only by comprehensively controlling the three reaction conditions.

#### 3.2.2. FT-IR Spectrum Analysis of Different SLPs

Figure 1(c) shows the FT-IR spectra of SLPs with different –OSO_3_^–^ contents. The broad absorption peak around 3385 cm^−1^ is the stretching vibration of O–H. The peak at 2931 cm^−1^ is the –CH stretching vibration of the –CH_3_ or –CH_2_ group. The peak at 1640 cm^−1^ is the symmetric or asymmetric stretching vibration of C=O. The peak at 1024 cm^−1^ is the stretching vibration of C–O. The infrared characteristic absorption peaks of SLPs with different –OSO_3_^–^ contents are shown in Table 2.

Compared with LP0, SLPs have two new peaks at 1259 and 820 cm^−1^, which are the asymmetric stretching vibration of S=O and stretching vibration of C–O–S, respectively [29]. The intensities of the two peaks gradually increase with –OSO_3_^–^ content in the polysaccharides (Figure 1(d)), that is, LP0 < SLP1 < SLP2 < SLP3 < SLP4. This result indicates successful sulfation modification.

#### 3.2.3. ^1^H NMR Spectrum Analysis

Figures 1(e) and 1(f) are the ^1^H NMR spectra of LP0 and SLP2, respectively. The D_2_O solvent peak is around the chemical shift (*δ*) of 4.71 ppm. The signal peak of the ring proton (H2–H5) is 3.6–4.7 ppm, and the methyl proton H-6 is approximately 1.3 ppm [30].

In the ^1^H NMR spectrum of LP0, *δ* 5.33, 4.90, 4.67, 3.68, 3.57, and 1.12 ppm are attributable to the chemical shift from H-1 to H-6 of (1 → 2,4)-*β*-D-Malp; *δ* 5.30, 3.96, 3.58, 3.62, 3.20, and 1.10 ppm are attributable to the chemical shifts from H-1 to H-6 of (1 → 6)-*α*-D-Malp; *δ* 5.15, 3.51, 3.78, 3.37, 4.11, and 3.82 ppm correspond to the chemical shifts from H-1 to H-6 of (1 → 4)-*α*-D-Glcp; *δ* 4.59, 3.35, 3.70, 3.89, 3.91, and 3.87 ppm are attributable to the chemical shifts from H-1 to H-6 of (1 → 6)-*β*-D-Glcp (Table 3) [31].

A new strong signal peak at *δ* 4.22 ppm was detected in the ^1^H NMR spectrum of SLP2 after sulfation modification. The appearance of the peak is attributable to the change of chemical shift caused by the introduction of the –OSO_3_^–^ group after sulfation [32]. The signal peak at *δ* 3.96 ppm shifts to *δ* 4.22 ppm, indicating that the hydroxyl group of SLP2 is sulfated. The signal peak of SLP2 at *δ* 1.10 ppm is significantly enhanced because of the substitution of the hydroxyl group by the –OSO_3_^–^ group after the sulfation of the polysaccharide.

#### 3.2.4. ^13^C NMR Spectrum Analysis

The ^13^C NMR spectra of LP0 and SLP2 are shown in Figures 1(g) and 1(h). Multiple strong signal peaks were detected in the anomeric proton (*δ* 93–106 ppm) and high field (*δ* 16.5–18.5 ppm) regions [33]. The peaks at *δ* 99.99, 76.68, 80.45, 57.41, 69.30, and 16.77 ppm belong to the signal peaks from C-1 to C-6 of (1 → 2,4)-*β*-D-Malp. The peaks at *δ* 99.60, 74.55, 70.36, 60.71, 62.67, and 16.65 ppm are attributable to the signal peaks from C-1 to C-6 of (1 → 6)-*α*-D-Malp. The peaks at *δ* 95.75, 71.51, 72.69, 72.85, 71.71, and 60.45 ppm are, respectively, attributable to the signal peaks from C-1 to C-6 of (1 → 4)-*α*-D-Glcp. The peaks at *δ* 100.27, 76.54, 78.70, 79.84, 73.33, and 71.17 ppm correspond to the signal peaks from C-1 to C-6 of (1 → 6)-*β*-D-Glcp (Table 3) [31].

The ^13^C NMR spectrum of SLP2 has the following changes relative to that of LP0:

1. The high field signal peak of SLP2 at *δ* 71.46 ppm is weakened, and a new peak appears at *δ* 76.20 ppm. The reason is the movement of carbon directly attached to the electron-withdrawing group (–OSO_3_^–^ group) after sulfation to a lower field position [34]. This movement indicates that the hydroxyl group at C-2 is sulfated
2. SLP2 shows signal peak splitting at *δ* 95–100 ppm. The functionalization of the –OH group on C-2 causes the signal peak of C-1 to split, and this split has a good correlation with the substitution degree of the C-2 atom [35], which further shows that the hydroxyl group on C-2 is sulfated
3. The signal peaks of SLP2 at *δ* 60.72 and 57.42 ppm are enhanced possibly because of the substitution of the hydroxyl group on C-4 by –OSO_3_^–^
4. The new signal peak of SLP2 at *δ* 66.93 ppm is attributable to O-6 substituted carbon, indicating that O-6 is sulfated [36]. The peak of SLP2 at *δ* 16.77 ppm is significantly enhanced, indicating that the hydroxyl group at the C-6 position in the SLPs is replaced by –OSO_3_^–^

According to the above analysis and reference [37], the –OH on C2, C4, and C6 of the original LP0 are replaced by –OSO_3_^–^ after the sulfation reaction (Figure 1(i)).

### 3.3. SLPs Regulate CaOx Crystallization

#### 3.3.1. XRD Analysis of CaOx Crystal

**Figure 2:**
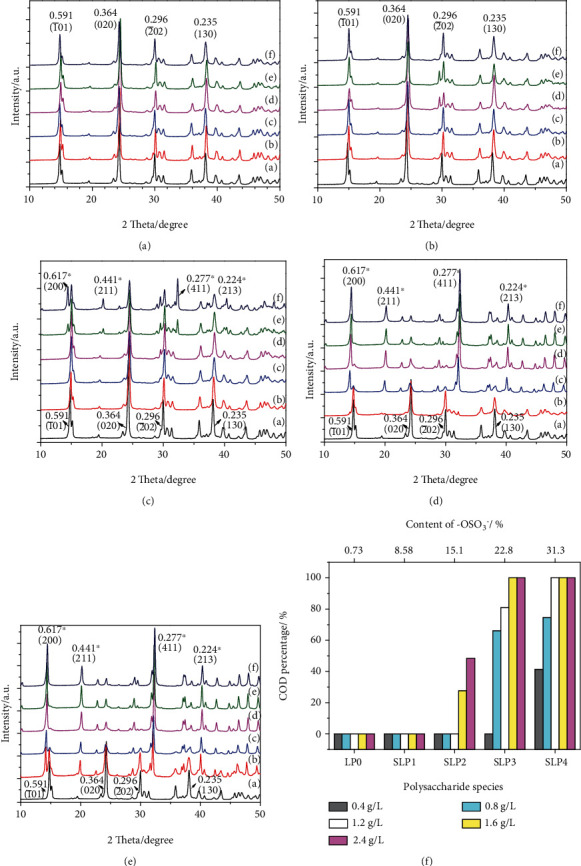
XRD spectra of CaOx crystals induced by SLPs at different concentrations. (a) LP0. (b) SLP1. (c) SLP2. (d) SLP3. (e) SLP4. (f) The percentage of COD in CaOx crystals formed in the presence of SLPs at various concentrations. Polysaccharide concentration in Figs.: (a) 0; (b) 0.4; (c) 0.8; (d) 1.2; (e) 1.6; (f) 2.4 g/L. Those marked with ^∗^ are COD diffraction peaks, and those without ^∗^ are COM diffraction peaks.

Figures 2(a)–2(e) are the XRD spectra of CaOx crystals induced by LP0 and four SLPs with different sulfation degrees. The diffraction peaks at interplanar spacing (d) values of 0.591, 0.364, 0.296, and 0.235 nm are attributed to the ($\bar{1}01$), (020), ($\bar{2}02$), and (130) crystal planes of COM, respectively. The diffraction peaks at d values of 0.617, 0.441, 0.277, and 0.224 nm are attributed to the (200), (211), (411), and (213) crystal planes of COD, respectively.

1. In LP0 and SLP1, no COD diffraction peak appears at all concentration ranges (0.40–2.40 g/L), indicating that they only form COM crystals
2. In SLP2 and SLP3, when the concentration is low (0.4 g/L), only COM crystals form (Figures 2(c) and 2(d)). When the concentration of SLP2 increases to 1.6 g/L, COD crystals form. When the concentration of SLP3 increases to 0.8 g/L, COD crystals appear. As the concentration of polysaccharides continues to increase, the percentage of COD in the crystals gradually increases. When the concentration of SLP3 increases to 1.6 g/L, 100% COD crystals are induced (Figure 2(f)). In the SLP4 with the highest –OSO_3_^–^ content, 41.2% COD formation is induced at 0.4 g/L, and 100% COD formation is induced at 1.2 g/L
3. At a concentration of 1.20 g/L, LP0, SLP1, SLP2, SLP3, and SLP4 induce 0, 0, 0, 81.0%, and 100% COD crystals, respectively. At a concentration of 2.40 g/L, the percentages of COD are 0, 0, 48.3%, 100%, and 100%, respectively

#### 3.3.2. FT-IR Spectrum

**Figure 3:**
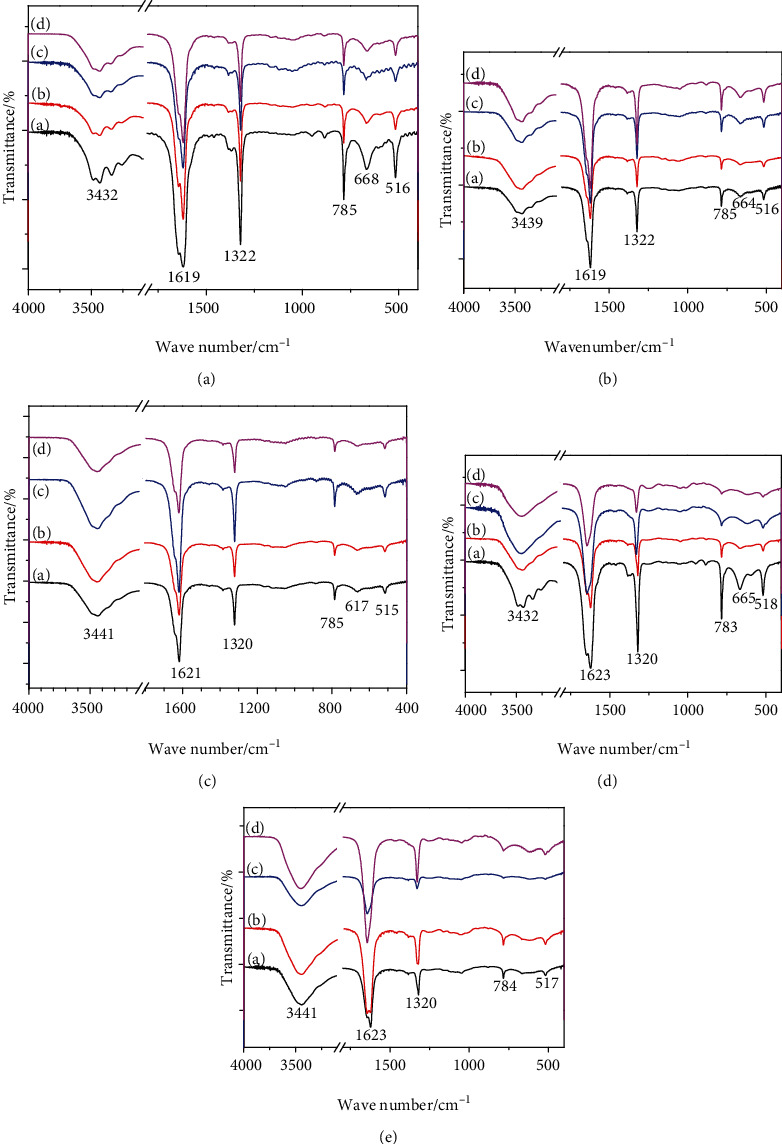
FT-IR spectra of CaOx crystals induced by SLPs at different concentrations. (a) LP0. (b) SLP1. (c) SLP2. (d) SLP3. (e) SLP4. In Figs. (a) 0.4; (b) 0.8; (c) 1.2; (d) 1.6 g/L.

Figure 3 shows the FT-IR spectra of CaOx crystals induced by SLPs at different concentrations. In LP0 and SLP1, the absorption peaks of induced crystals in all concentration ranges (0.4–1.6 g/L) are similar (Figures 3(a) and 3(b)), and five stretching vibration peaks at 3000–3600 cm^−1^ belong to the O–H bond of COM crystal water. The asymmetric stretching vibration *ν*_as_(COO^−^) and the symmetric stretching vibration *ν*_s_(COO^−^) of the carboxyl group are near 1619 and 1322 cm^−1^, respectively (Table 4), indicating that only the COM crystal is formed [38, 39].

SLP2, SLP3, and SLP4 mainly induce the formation of COM at low concentrations and also induce COD crystal formation at high concentrations. The FT-IR spectrum of COD differs from that of COM. COD crystal has only one strong and broad absorption peak in the 3000–3600 cm^−1^ region, and *ν*_as_(COO^−^) and *ν*_s_(COO^−^) are at 1646 and 1330 cm^−1^, respectively (Table 4) [38, 39].

Comprehensive XRD and FT-IR results show that the ability of SLPs to induce COD is positively correlated with the –OSO_3_^−^ content of polysaccharides. Increase in the –OSO_3_^−^ content or concentration of polysaccharides inhibits the formation of COM and induces the formation of COD.

#### 3.3.3. SEM Observation of CaOx Crystal

**Figure 4:**
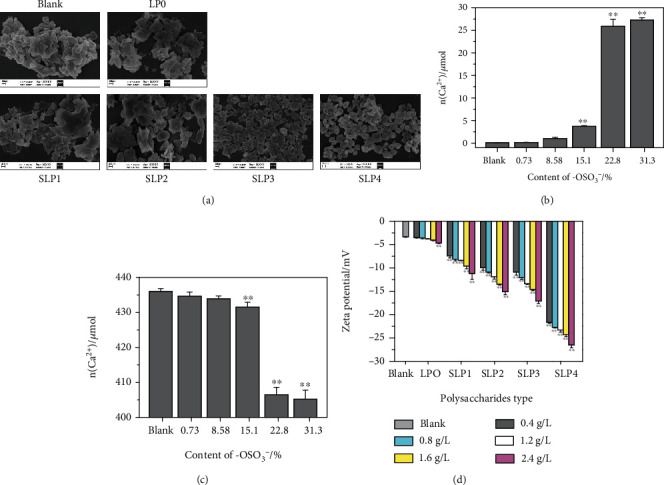
(a) SEM images of CaOx crystals, (b) soluble Ca^2+^ ions concentration in the supernatant, and (c) CaOx precipitate amount in the presence of SLPs at 1.6 g/L. (d) The influence of SLPs on the Zeta potential of CaOx crystals formed. Compared with the blank group, ^∗^*P* < 0.05; ^∗∗^*P* < 0.01.

Figure 4(a) shows the SEM images of each SLP that induces the formation of CaOx crystals at 1.6 g/L. The crystals formed in the blank group have a strong three-dimensional effect, and serious aggregation phenomena occur. Compared with the crystals in the blank group, the crystals regulated by LP0 and SLP1 gradually transform into lamellae, but the degree of aggregation is weakened. XRD results show that the crystals formed in the blank, LP0, and SLP1 groups are COM crystals.

COD crystals appear in SLP2-regulated crystals (approximately 27.7%, Figure 2(f)). Crystal size regulated by SLP3 and SLP4 is further reduced, the degree of aggregation between the crystals is further reduced, and the percentage of tetragonal bipyramid COD crystals is significantly increased. That is, an increase in –OSO_3_^−^ content of polysaccharide reduces the size and aggregation degree of CaOx crystals and induces the formation of COD crystals.

#### 3.3.4. Detection of Soluble Ca^2+^ Ion Concentration and CaOx Precipitation in the System

The soluble Ca^2+^ ion concentration in each SLPs system at 1.6 g/L was detected by ICP, and the mass of each CaOx crystal formed was weighed. As the –OSO_3_^–^ contents of the SLPs increase, the concentration of soluble Ca^2+^ ions in the supernatant gradually increases (Figure 4(b)), whereas the amount of CaOx precipitates gradually decreases (Figure 4(c)).

For example, the masses of CaOx precipitates induced by LP0 and SLP4 are 63.5 and 66.5 mg, respectively, and the corresponding substance amounts are 434.6 and 405.2 *μ*mol. Soluble Ca^2+^ concentrations in the supernatant are 0.53 and 101.7 mg/L, and the substance amounts are 0.16 and 27.26 *μ*mol, respectively. This is because COD has one more water molecule than COM, which increases its relative molecular mass, so the mass of the precipitate increases with the same amount of substance (Table 5).

#### 3.3.5. Zeta Potential of CaOx Crystal

Zeta potential can be used in measuring the amount of repulsive force between crystals. When the absolute value of the zeta potential increases, the strength of the repulsive force between crystals and the dispersion of crystals in a solution increases, and the aggregation of crystals is inhibited [40].

Figure 4(d) shows the absolute value of the Zeta potential of CaOx crystals produced by the regulation of five different SLPs with different –OSO_3_^−^ contents. The absolute value of the zeta potential of the CaOx crystals produced by the regulation gradually increases. At the same concentration, as the –OSO_3_^−^ content of polysaccharide increases, the absolute value of the crystal's Zeta potential gradually increases. For example, when the polysaccharide concentration is 2.4 g/L, the absolute values of the zeta potential of the CaOx crystals generated have the following order: LP0 (4.67 mV) < SLP1 (11.2 mV) < SLP2 (15.1 mV) < SLP3 (17.1 mV) < SLP4 (26.5 mV).

### 3.4. In Vitro Antioxidant Capacity of SLPs with Different –OSO_3_^−^ Content

On the basis of the tested ability of SLPs with different –OSO_3_^–^ contents to scavenge hydroxyl (•OH) free radicals and DPPH free radicals, their in vitro antioxidant activities were compared. The –OSO_3_^–^ group in SLPs can inhibit the generation of hydroxyl radicals by chelating Fe^2+^ [41]. In addition, when SLPs interact with DPPH, they transfer electrons or hydrogen atoms to DPPH, thereby neutralizing its free radicals [42].

**Figure 5:**
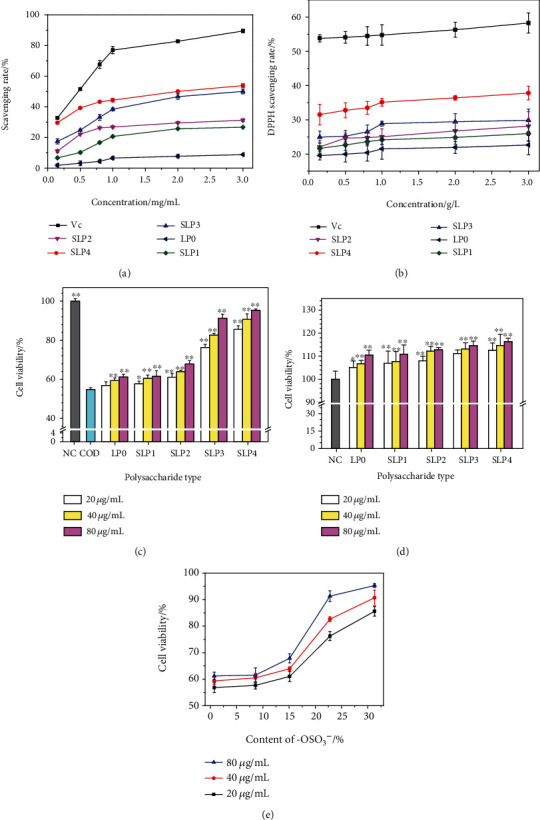
In vitro antioxidant capacity of SLPs. (a) Scavenging •OH free radicals; (b) Scavenging DPPH free radicals. Comparison of the ability of SLPs to protect HK-2 cells from oxidative damage. (c) Cell viability was detected by the CCK-8 method. Compared with the COD group, ^∗^*P* < 0.05; ^∗∗^*P* < 0.01. (d) Cell cytotoxicity was detected by the CCK-8 method. Compared with the NC group, ^∗^*P* < 0.05; ^∗∗^*P* < 0.01. (e) The relationship between cell viability and the –OSO_3_^−^ content of polysaccharide. NC: normal control; COD concentration: 200 *μ*g/mL; damage time: 12 h; protection time: 12 h.

Figures 5(a) and 5(b) shows the ability of SLPs with different –OSO_3_^–^ content to scavenge •OH radicals and DPPH radicals and their concentration effects. As the concentration of polysaccharides increases, the ability to scavenge •OH free radicals and DPPH free radicals increases, that is, the antioxidant capacities of polysaccharides are concentration dependent. Notably, a polysaccharide concentration of 1.0 mg/mL is a turning point for improving the speed of scavenging free radicals. When the polysaccharide concentration is less than 1.0 mg/mL, the ability of polysaccharides to scavenge free radicals increases rapidly with polysaccharide concentration. When the polysaccharide concentration is greater than 1.0 mg/mL, the ability of polysaccharides to scavenge free radicals increases slowly with increasing polysaccharide concentration.

At the same concentration, as the –OSO_3_^–^ contents of polysaccharides increase, the ability of the polysaccharides to scavenge free radicals increases, that is, the antioxidant capacities of SLPs are positively correlated with the content of –OSO_3_^–^ in SLPs. In •OH free radicals, SLP3 and SLP4 have a significantly stronger free radical scavenging ability than other polysaccharides. For DPPH free radicals, SLP4 with a high –OSO_3_^–^ content has a significantly stronger free radical scavenging ability than other polysaccharides.

### 3.5. Comparison of the Ability of SLPs with Different -OSO_3_^−^ Content to Protect HK-2 Cells from Oxidative Damage

#### 3.5.1. Cell Viability

After HK-2 cells are damaged by 200 *μ*g/mL nano-COD crystals for 12 h, cell viability decreases from 100.00% ± 1.27% to 54.84% ± 0.85%, indicating that COD crystals have obvious damage to cells, and this damage was moderate, which was convenient to carry out the protection experiment of polysaccharide [14, 43]. The five polysaccharides used three concentrations of 20, 40, and 80 *μ*g/mL to protect HK-2 cells. Cell viability test results show that cell viability increases compared with that in the nano-COD damage group because of the protection of HK-2 cells by the polysaccharides at each concentration, and each polysaccharide had the best protective effect at 80 *μ*g/mL.

The protective effect of each SLP on HK-2 cells was compared at the same concentration (Figure 5(c)), and the results show that the degree of protection against nano-COD crystal damage in the HK-2 cells increases with the polysaccharide –OSO_3_^–^ content. At the best protective concentration (80 *μ*g/mL), the cell viability of the five polysaccharide protection groups is 61.24% (LP0), 61.54% (SLP1), 67.87% (SLP2), 91.30% (SLP3), and 95.31% (SLP4).

#### 3.5.2. Cytotoxicity of Polysaccharides

As shown in Figure 5(d), different concentrations of SLPs interact with cells for 24 h. Cell viability is greater than the normal group (100%), indicating that the polysaccharides are not toxic to cells at 20–80 *μ*g/mL and can promote cell growth. SLP4 with the highest –OSO_3_^–^ content has the strongest effect on HK-2 cell proliferation.

#### 3.5.3. Reactive Oxygen Species (ROS) Level

**Figure 6:**
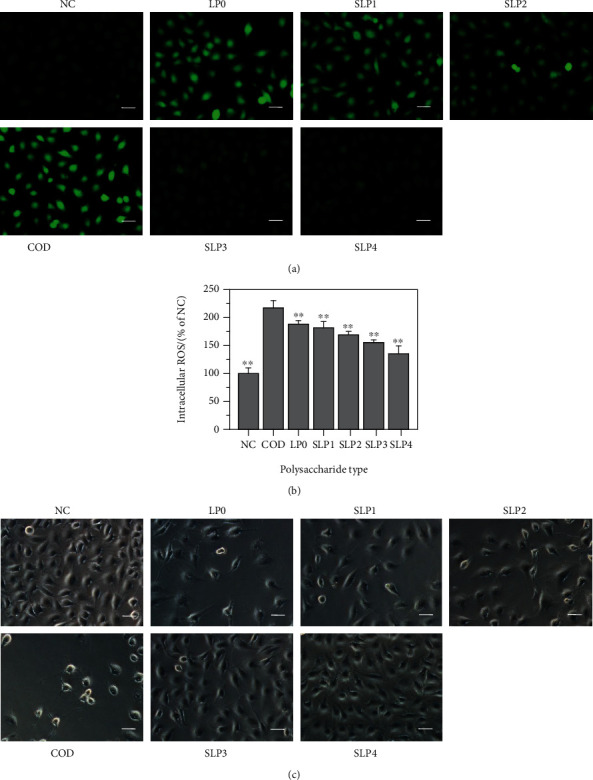
ROS level and cell morphology were detected before and after SLPs protect HK-2 cells. (a) ROS fluorescence microscope images. (b) Quantitative histogram of ROS fluorescence intensity. (c) Cell morphology. NC: normal control; polysaccharide concentration: 80 *μ*g/mL; COD concentration: 200 *μ*g/mL; damage time: 12 h; protection time: 12 h. Scale bars: 50 *μ*m. Compared with the COD group, ^∗^*P* < 0.05; ^∗∗^*P* < 0.01.

A large amount of ROS is produced in damaged cells, resulting in abnormal cell function and even cell death [44].  Figure 6(a) shows the ROS fluorescence intensity of each group of cells. The intensities were detected using the DCFH-DA fluorescent probe method. The ROS fluorescence intensity in the normal group is the lowest. The ROS fluorescence intensity increases significantly after normal cells are damaged by 200 nm COD. After the addition of four kinds of polysaccharides, ROS fluorescence intensity decreases. As the –OSO_3_^–^ content of polysaccharide increases, the ROS fluorescence intensity gradually decreases. The highest degree decrease in ROS fluorescence intensity was observed after the addition of SLP4 (Figure 6(b)), owing to the protective effect of the polysaccharide. This result shows that SLPs can protect cells from damage caused by nano-COD crystals, and the protective ability of polysaccharides is positively correlated with the –OSO_3_^–^ contents of polysaccharides.

#### 3.5.4. Cell Morphology

As shown in Figure 6(c), the cell in the normal group is evenly distributed, the cell morphology is full, and the cells are tightly connected. After the normal cells are damaged by 200 nm COD, the number of cells is significantly reduced, cell shape shrinks, and the connections between cells are destroyed. After the addition of various polysaccharides, the degree of damage caused by COD on the cells is reduced, the number of cells in the protection groups is higher than that in the damage group, the connection between cells is gradually restored, and the cell morphology is significantly improved. The ability of the polysaccharides to protect HK-2 cells from COD damage increases with –OSO_3_^–^ content of polysaccharide. The cell morphology of SLP4 with the highest –OSO_3_^–^ content is closest to the normal group.

## 4. Discussion

### 4.1. Sulfation and Structural Analysis of SLPs

Sulfation of polysaccharides can change the biological activity and function of natural products [45]. The sulfur trioxide-pyridine method is a common sulfation method. This paper uses this method to sulfate LP0 with an initial –OSO_3_^–^ content of 0.73%. By controlling the reaction time, reactant (sulfur trioxide-pyridine complex) concentration, and reaction temperature, four kinds of modified polysaccharides were obtained. The –OSO_3_^–^ contents are 8.58% (SLP1), 15.1% (SLP2), 22.8% (SLP3), and 31.3% (SLP4). The results of FT-IR, ^1^H NMR, and ^13^C NMR spectroscopy show that sulfation causes no change in the monosaccharide composition of polysaccharides. They are all composed of maltose and glucose with traces of sucrose, fructose and raffinose (Figures 1(a) and 1(b)). The main sugar residues are (1 → 2,4)-*β*-D-Malp, (1 → 6)-*α*-D-Malp, (1 → 4)-*α*-D-Glcp, and (1 → 6)-*β*-D-Glcp unit.

### 4.2. Enhanced Antioxidant Capacities of Sulfated SLPs

Compared with LP0, sulfated SLPs have a better scavenging ability for •OH and DPPH radicals. The reason is that the introduction of –OSO_3_^–^ groups weakens the dissociation energy of O–H bonds in polysaccharide molecules [45] and improves the hydrogen supply capacities of polysaccharide derivatives. Hydrogen atoms provided by polysaccharides combine with free radicals to form stable free radicals and terminates free radical chain reaction, thereby increasing antioxidant activity [46, 47]. For example, after polysaccharides donate hydrogen atoms or single electrons to DPPH, nonradical compounds DPPH-H are generated [48]. Huang et al. [49] modified Mesona chinensis Benth polysaccharide through sulfation to obtain SMP. The scavenging rates of the two on DPPH free radicals are 75.11% and 86.95%, respectively, indicating that increase in the –OSO_3_^–^ contents of polysaccharides improve antioxidant activity. Hu et al. [50] modified Acanthopanax leucorrhizus polysaccharide (ALP) by sulfation to obtain S-ALP1 and S-ALP2 components with the substitution degrees of 0.48 and 0.73, respectively. The scavenging ability of the three on hydroxyl radicals is S-ALP2 > S-ALP1 > ALP, indicating that the antioxidant activity is positively correlated with the –OSO_3_^–^ content of polysaccharide, that is, the higher the –OSO_3_^–^ content of the polysaccharide, the stronger the antioxidant activity.

### 4.3. Sulfated SLPs Have a Stronger Ability to Regulate CaOx Crystallization

Compared with the original LP0, the sulfated SLPs can better inhibit the formation of CaOx crystals (Figures 2–4), due to the following reasons.

First, the sulfated SLPs are rich in acidic –OSO_3_^–^ groups, which can better combine with the free Ca^2+^ ions in the solution to form soluble complexes, increase the soluble Ca^2+^ ion concentration in the system (Figure 4(b)), and reduce the amount of Ca^2+^ ions combined with Ox^2-^, thereby reducing the amount of CaOx precipitation, which inhibit the formation of CaOx stones [51].

Second, SLPs form polyanions in the solution and are adsorbed on the surfaces of CaOx crystals, causing defects in crystal growth, preventing free particles from entering, and inhibiting the growth of CaOx crystals. Melo et al. [52] extracted four sulfated polysaccharide components from the marine alga Dictyopteris justii. Their –OSO_3_^–^ content was 3.9%, 4.3%, 6.8%, and 7.5%, respectively. Among them, the component with the highest -OSO_3_^−^ content has the best effect on inhibiting CaOx crystallization.

**Figure 7:**
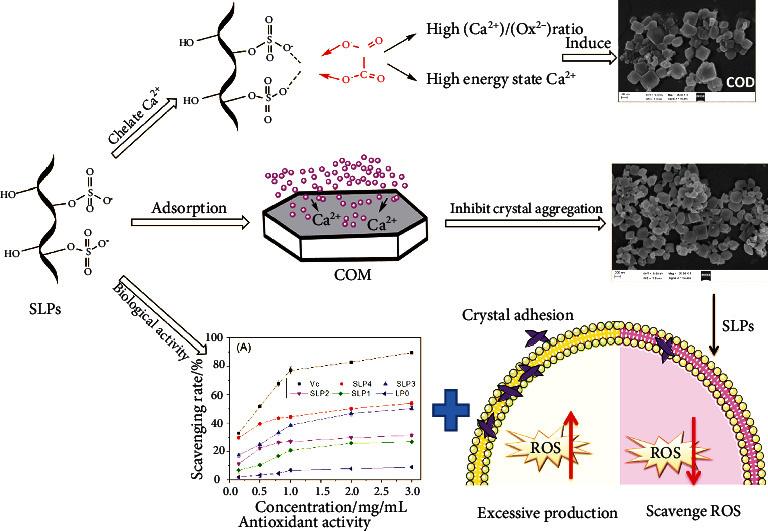
The mechanism diagram of SLPs inhibiting kidney stones formation.

Third, SLPs with a high –OSO_3_^–^ content induce COD crystal formation. The reason is as follows: SLPs rich in acidic –OSO_3_^–^ groups can adsorb a large amount of Ca^2+^ ions through electrostatic attraction, enriching the Ca^2+^ ions on and near the polysaccharide surfaces and increasing [Ca^2+^]/[Ox^2-^] molar ratio. Additionally, the energy interfaces of the polysaccharide molecule surfaces increase. The ratio of adsorbed Ca^2+^ ions leads to an increase in the energy state of free Ca^2+^ ions. The high-energy interface and high-energy state Ca^2+^ ions promote the formation of thermodynamic metastable COD [53].  Figure 7 shows the mechanism of SLPs inhibiting kidney stones formation.

Notably, SLP3 with –OSO_3_^–^ content of 22.8% is a turning point in the mutation of a polysaccharide effect, that is, the ability of SLP3 and SLP4 to regulate the formation of COD crystals and their antioxidant activity and ability to protect cells from COD crystals are significantly greater than those of LP0, SLP1, and SLP2.

Compared with COD, COM crystals are difficult to excrete from the body because of their stronger adhesion to damaged renal epithelial cells. Therefore, sulfated SLPs that induce COD crystals are more useful in inhibiting the formation of CaOx stones than the original LP0.

SLPs with high –OSO_3_^–^ contents can inhibit the aggregation of CaOx crystals (Figure 4(a)). Aggregated crystals are not only difficult to excrete from the body but also cause considerable damage to renal epithelial cells [54, 55], thereby increasing the risk of kidney stone formation. Figure 4(d) shows that the absolute value of the zeta potential of CaOx crystals generated by the regulation of sulfated SLPs is much higher than that of the original polysaccharide LP0. The surface charge density of a crystal and the amount of repulsive force between crystals increase with the absolute value of the zeta potential on the crystal surface, and the aggregation of crystals is inhibited [56].

### 4.4. Sulfated SLPs Have a Strong Ability to Protect HK-2 Cells from Crystal Damage

The biological activity of sulfated polysaccharides is closely related to their –OSO_3_^–^ content. Compared with LP0, sulfated SLPs can better protect HK-2 cells from the damage of nano-COD crystals. The results of this paper show that the ability of SLPs to protect cells from crystals damage is positively correlated with their –OSO_3_^–^ content (Figure 5(e)). As the –OSO_3_^–^ content of SLPs increases, cell viability gradually increases (Figure 5(c)), ROS levels decrease (Figure 6(a)), and cell morphology gradually recovers (Figure 6(c)). The ability of SLP3 and SLP4 to protect cells from COD crystal damage is significantly greater than that of LP0, SLP1, and SLP2.

Jin et al. [57] found that sulfation modification can improve the antioxidant activity of polysaccharides and can better protect cells from oxidative damage and apoptosis induced by H_2_O_2_. Wang et al. [58] showed that compared with the original *Cyclocarya paliurus* polysaccharides, sulfated polysaccharides have a better protective effect on oxidative stress caused by H_2_O_2_. Moreover, Wang et al. [59] proved that sulfation modification can enhance the immune activity of *Lycium barbarum* polysaccharide.

## 5. Conclusions

In this study, the original LP0 was sulfated using the sulfur trioxide-pyridine method, and four kinds of sulfated polysaccharide SLPs with the –OSO_3_^−^ contents of 8.58%, 15.1%, 22.8%, and 31.3% were obtained. As the -OSO_3_^−^ content of polysaccharides increases, its regulating effect on the growth of CaOx crystals is enhanced. As a result, the concentration of soluble Ca^2+^ in the solution increases, the amount of CaOx crystal precipitation is reduced, the degree of CaOx crystal aggregation is significantly reduced, and the percentage of induced COD crystals increases. These effects can effectively inhibit the formation of CaOx kidney stones. The in vitro antioxidant activity of SLPs is positively correlated with their –OSO_3_^−^ content. SLPs are not toxic to HK-2 cells. The level of their ability to protect cells from damage by nano-COD crystals increases with –OSO_3_^−^ content. Sulfation modification can improve the biological activities of polysaccharides, providing a good prospect for finding and developing effective drugs for treating kidney stones.

## Figures and Tables

**Table 1:** Sulfation conditions of five SLPs with different -OSO_3_^−^ content.

SLPs	Sulfation conditions			–OSO_3_^–^ content/%
	Reaction time/h	Sulfur trioxide-pyridine complex/g	Reaction temperature/°C	
LP0	—	—	—	0.73
SLP1	0.5	1	40	8.58
SLP2	0.5	2	40	15.1
SLP3	2	5	60	22.8
SLP4	8	5	60	31.3

**Table 2:** FT-IR characteristic absorption peaks of SLPs with different –OSO_3_^−^ content.

SLPs	–OSO_3_^–^content/%	Characteristic absorption peak/cm^−1^					
		O-H	-CH_2_-	C=O	C-O	–OSO_3_^–^	
						S=O	C-O-S
LP0	0.73	3385	2931	1640	1024	/	/
SLP1	8.58	3416	2932	1636	1024	1252	817
SLP2	15.1	3418	2932	1640	1022	1254	820
SLP3	22.8	3424	2953	1642	1007	1258	821.5
SLP4	31.3	3424	2957	1640	1008	1259	822

**Table 3:** Chemical shifts in ^1^H NMR and ^13^C NMR spectra of LP0 and SLP2 (*δ*).

SLPs	Sugar residue	Chemical shift (ppm)					
		H-1/C-1	H-2/C-2	H-3/C-3	H-4/C-4	H-5/C-5	H-6/C-6
LP0	(1 → 2,4)-*β*-D-Malp	5.33/99.99	4.90/76.68	4.67/80.45	3.68/57.41	3.57/69.30	1.12/16.77
	(1 → 6)-*α*-D-Malp	5.30/99.60	3.96/74.55	3.58/70.36	3.62/60.71	3.20/62.67	1.10/16.65
	(1 → 4)-*α*-D-Glcp	5.15/95.75	3.51/71.51	3.78/72.69	3.37/72.85	4.11/71.71	3.82/60.45
	(1 → 6)-*β*-D-Glcp	4.59/100.27	3.35/76.54	3.70/78.70	3.89/79.84	3.91/73.33	3.87/71.17

SLP2	(1 → 2,4)-*β*-D-Malp	5.33/99.64	4.90/76.65	4.68/82.69	3.69/57.42	3.57/69.31	1.11/16.77
	(1 → 6)-*α*-D- Malp	5.34/99.60	4.22/74.55	3.58/70.32	3.62/60.72	3.20/63.03	1.10/16.65
	(1 → 4)-*α*-D-Glcp	5.15/95.75	3.56/71.46	3.78/72.67	3.36/72.85	4.11/71.65	3.82/60.46
	(1 → 6)-*β*-D-Glcp	4.57/100.10	3.34/76.20	3.70/76.85	3.89/79.75	3.91/73.33	3.84/71.14

^∗^Malp: maltose; Glcp: glucose.

**Table 4:** Infrared characteristic absorption peaks of CaOx crystals regulated by different concentrations of SLPs.

SLPs	c(SLPs)/g/L	COD/%	*ν*_as_(COO^−^)/cm^−1^	*ν*_as_(COO^−^)/cm^−1^	*ν*_s_(COO^−^)/cm^−1^	COM/cm^−1^	COM/cm^−1^	COM/cm^−1^	COM/cm^−1^	COD /cm^−1^	COD /cm^−1^
Blank	0	0		1619	1319	950	882	785	668		

LP0	0.4	0		1619	1319	950	882	785	668		
	0.8	0		1620	1319	950	882	785	668		
	1.2	0		1620	1319	951	882	785	668		
	1.6	0		1619	1319	951	883	785	663		

SLP1	0.4	0		1619	1319	952	882	785	664		
	0.8	0		1619	1320		881	785	664		
	1.2	0		1619	1320	948	881	785	667		
	1.6	0		1619	1320	950	882	785	668		

SLP2	0.4	0		1619	1320	951	883	785	662		
	0.8	0		1619	1320	952	881	785	665		
	1.2	0		1620	1320	951	883	785	668		
	1.6	27.7		1621	1321	952	882	785	660		

SLP3	0.4	0		1623	1320	950	882	784	665		
	0.8	66.1		1623	1323	948	881	784	664		617
	1.2	81.0	1645		1328			784		915	618
	1.6	100	1646		1329					917	619

SLP4	0.4	41.2		1625	1322		881	784	668		
	0.8	74.6	1645		1324			784		914	620
	1.2	100	1646		1330					913	621
	1.6	100	1646		1330					919	619

**Table 5:** The crystal phase of calcium oxalate crystals, the amount of precipitation and the concentration of soluble Ca^2+^ ions in the supernatant formed in the presence of 1.6 g/L SLPs.

SLPs	c(SLPs)/g/L	COD/%	c(Ca^2+^)/mg/L	n(Ca^2+^)/*μ*mol	m(CaOx) /mg	n(CaOx)/*μ*mol	n(total Ca^2+^)/*μ*mol
Blank	—	0	0.35	0.11	63.7	436.0	436.1
LP0 (0.73%)	1.6	0	0.53	0.16	63.5	434.6	434.8
SLP1 (8.58%)	1.6	0	3.47	1.05	63.4	433.9	435.0
SLP2 (15.1%)	1.6	27.7	12.84	3.74	65.2	431.5	435.2
SLP3 (22.8%)	1.6	100	95.58	25.90	66.7	406.5	432.4
SLP4(31.3%)	1.6	100	101.7	27.26	66.5	405.2	432.5

## Data Availability

All the data supporting the results were shown in the paper and can be applicable from the corresponding author.

## References

[1] Wang W. Y., Fan J. Y., Huang G. F. (2017). Prevalence of kidney stones in mainland China: a systematic review. *Scientific Reports*.

[2] Khamchun S., Sueksakit K., Chaiyarit S., Thongboonkerd V. (2019). Modulatory effects of fibronectin on calcium oxalate crystallization, growth, aggregation, adhesion on renal tubular cells, and invasion through extracellular matrix. *Journal of Biological Inorganic Chemistry*.

[3] Mirković M., Dosen A., Erić S., Vulić P., Matović B., Rosić A. (2020). Phase and microstructural study of urinary stones. *Microchemical Journal*.

[4] Parvaneh L. S., Donadio D., Sulpizi M. (2016). Molecular mechanism of crystal growth inhibition at the calcium oxalate/water interfaces. *The Journal of Physical Chemistry C*.

[5] de Bellis R., Piacentini M. P., Meli M. A. (2019). In vitro effects on calcium oxalate crystallization kinetics and crystal morphology of an aqueous extract from ceterach officinarum: analysis of a potential antilithiatic mechanism. *PLoS One*.

[6] Chung J., Granja I., Taylor M. G., Mpourmpakis G., Asplin J. R., Rimer J. D. (2016). Molecular modifiers reveal a mechanism of pathological crystal growth inhibition. *Nature*.

[7] Unno R., Kawabata T., Taguchi K. (2020). Deregulated MTOR (mechanistic target of rapamycin kinase) is responsible for autophagy defects exacerbating kidney stone development. *Autophagy*.

[8] Huang L. S., Sun X. Y., Gui Q., Ouyang J. M. (2017). Effects of plant polysaccharides with different carboxyl group contents on calcium oxalate crystal growth. *CrystEngComm*.

[9] Gangu K. K., Tammineni G. R., Dadhich A. S., Mukkamala S. B. (2014). Control of phase and morphology of calcium oxalate crystals by natural polysaccharide, gum Arabic. *Molecular Crystals and Liquid Crystals*.

[10] Khan S. R. (2014). Reactive oxygen species, inflammation and calcium oxalate nephrolithiasis. *Translational andrology and urology*.

[11] Qi S. Y., Wang Q., Xie B., Chen Y., Zhang Z. H., Xu Y. (2020). P 38 MAPK signaling pathway mediates COM crystal-induced crystal adhesion change in rat renal tubular epithelial cells. *Urolithiasis*.

[12] Chen W., Liu W. R., Hou J. B. (2019). Metabolomic analysis reveals a protective effect of Fu-fang-Jin-Qian-Chao herbal granules on oxalate-induced kidney injury. *Bioscience Reports*.

[13] Xi Q. L., Ouyang J., Pu J. X., Hou J. Q., Wang S. G. (2015). High concentration of calcium stimulates calcium oxalate crystal attachment to rat tubular epithelial NRK cells through osteopontin. *Urology*.

[14] Zhao Y. W., Liu L., Li C. Y., Zhang H., Sun X. Y., Ouyang J. M. (2020). Preprotection of tea polysaccharides with different molecular weights can reduce the adhesion between renal epithelial cells and nano-calcium oxalate crystals. *Oxidative Medicine and Cellular Longevity*.

[15] Chen Y., Zhang H., Wang Y. X., Nie S. P., Li C., Xie M. Y. (2015). Sulfated modification of the polysaccharides from Ganoderma atrum and their antioxidant and immunomodulating activities. *Food Chemistry*.

[16] Chen F., Huang G. L., Yang Z. Y., Hou Y. P. (2019). Antioxidant activity of Momordica charantia polysaccharide and its derivatives. *International Journal of Biological Macromolecules*.

[17] Lu X. Y., Guo H., Zhang Y. L. (2012). Protective effects of sulfated chitooligosaccharides against hydrogen peroxide-induced damage in MIN6 cells. *International Journal of Biological Macromolecules*.

[18] Wang J., Wang F., Yun H., Zhang H., Zhang Q. B. (2012). Effect and mechanism of fucoidan derivatives from Laminaria japonica in experimental adenine-induced chronic kidney disease. *Journal of Ethnopharmacology*.

[19] Wang J., Zhang Q. B., Zhang Z. S., Li A. E. (2008). Antioxidant activity of sulfated polysaccharide fractions extracted from Laminaria japonica. *International Journal of Biological Macromolecules*.

[20] Wang J., Liu L., Zhang Q. B., Zhang Z. S., Qi H. M., Li P. C. (2009). Synthesized oversulphated, acetylated and benzoylated derivatives of fucoidan extracted from Laminaria japonica and their potential antioxidant activity in vitro. *Food Chemistry*.

[21] Yoon S. J., Pyun Y. R., Hwang J. K., Mourão P. A. S. (2007). A sulfated fucan from the brown alga _Laminaria cichorioides_ has mainly heparin cofactor II-dependent anticoagulant activity. *Carbohydrate Research*.

[22] Cui C., Lu J. H., Sun-Waterhouse D. X. (2016). Polysaccharides from Laminaria japonica: Structural characteristics and antioxidant activity. *Lebensmittel-Wissenschaft & Technologie*.

[23] Saha S., Navid M. H., Bandyopadhyay S. S., Schnitzler P., Ray B. (2012). Sulfated polysaccharides from Laminaria angustata: Structural features and in vitro antiviral activities. *Carbohydrate Polymers*.

[24] Wang J. M., Sun X. Y., Ouyang J. M. (2018). Structural characterization, antioxidant activity, and biomedical application of astragalus polysaccharide degradation products. *International Journal of Polymer Science*.

[25] Bedini E., Laezza A., Parrilli M., Iadonisi A. (2017). A review of chemical methods for the selective sulfation and desulfation of polysaccharides. *Carbohydrate Polymers*.

[26] Li S., Shah N. P. (2014). Antioxidant and antibacterial activities of sulphated polysaccharides from Pleurotus eryngii and Streptococcus thermophilus ASCC 1275. *Food Chemistry*.

[27] Sakthivel R., Pandima Devi K. (2015). Evaluation of physicochemical properties, proximate and nutritional composition of Gracilaria edulis collected from Palk Bay. *Food Chemistry*.

[28] Seedevi P., Moovendhan M., Viramani S., Shanmugam A. (2017). Bioactive potential and structural chracterization of sulfated polysaccharide from seaweed (Gracilaria corticata). *Carbohydrate Polymers*.

[29] Chen L., Huang G. L. (2019). Antioxidant activities of sulfated pumpkin polysaccharides. *International Journal of Biological Macromolecules*.

[30] Kariya Y., Mulloy B., Imai K. (2004). Isolation and partial characterization of fucan sulfates from the body wall of sea cucumber Stichopus japonicus and their ability to inhibit osteoclastogenesis. *Carbohydrate Research*.

[31] Zha X. Q., Lu C. Q., Cui S. H. (2015). Structural identification and immunostimulating activity of a Laminaria japonica polysaccharide. *International Journal of Biological Macromolecules*.

[32] Chen S. G., Wang J. F., Xue C. H. (2010). Sulfation of a squid ink polysaccharide and its inhibitory effect on tumor cell metastasis. *Carbohydrate Polymers*.

[33] Wang J., Zhang Q. B., Zhang Z. S., Zhang H., Niu X. Z. (2010). Structural studies on a novel fucogalactan sulfate extracted from the brown seaweed Laminaria japonica. *International Journal of Biological Macromolecules*.

[34] Li J. X., Chi Z., Yu L. J., Jiang F., Liu C. G. (2017). Sulfated modification, characterization, and antioxidant and moisture absorption/retention activities of a soluble neutral polysaccharide from Enteromorpha prolifera. *International Journal of Biological Macromolecules*.

[35] Wang J. L., Guo H. Y., Zhang J. (2010). Sulfated modification, characterization and structure-antioxidant relationships of Artemisia sphaerocephala polysaccharides. *Carbohydrate Polymers*.

[36] Liang L., Ao L., Ma T. (2018). Sulfated modification and anticoagulant activity of pumpkin (Cucurbita pepo, Lady Godiva) polysaccharide. *International Journal of Biological Macromolecules*.

[37] Zargarzadeh M., Amaral A. J. R., Custódio C. A., Mano J. F. (2020). Biomedical applications of laminarin. *Carbohydrate Polymers*.

[38] Akın B., Öner M., Bayram Y., Demadis K. D. (2008). Effects of carboxylate-modified, “Green” inulin biopolymers on the crystal growth of calcium oxalate. *Crystal Growth & Design*.

[39] Akyol E., Öner M. (2014). Controlling of morphology and polymorph of calcium oxalate crystals by using polyelectrolytes. *Journal of Crystal Growth*.

[40] Inagawa A., Fukuyama M., Hibara A., Harada M., Okada T. (2018). Zeta potential determination with a microchannel fabricated in solidified solvents. *Journal of Colloid and Interface Science*.

[41] Wei D. F., Chen T., Yan M. F. (2015). Synthesis, characterization, antioxidant activity and neuroprotective effects of selenium polysaccharide from Radix hedysari. *Carbohydrate Polymers*.

[42] Lajili S., Ammar H. H., Mzoughi Z. (2019). Characterization of sulfated polysaccharide from Laurencia obtusa and its apoptotic, gastroprotective and antioxidant activities. *International Journal of Biological Macromolecules*.

[43] Zhang H., Sun X. Y., Chen X. W., Ouyang J. M. (2020). Degraded Porphyra yezoensis polysaccharide protects HK-2 cells and reduces nano-COM crystal toxicity, adhesion and endocytosis. *Journal of Materials Chemistry B*.

[44] Khan S. R. (2013). Reactive oxygen species as the molecular modulators of calcium oxalate kidney stone formation: evidence from clinical and experimental investigations. *Journal of Urology*.

[45] Xie J. H., Wang Z. J., Shen M. Y. (2016). Sulfated modification, characterization and antioxidant activities of polysaccharide from Cyclocarya paliurus. *Food Hydrocolloids*.

[46] Xu Y., Wu Y. J., Sun P. L., Zhang F. M., Linhardt R. J., Zhang A. Q. (2019). Chemically modified polysaccharides: synthesis, characterization, structure activity relationships of action. *International Journal of Biological Macromolecules*.

[47] Wang Z. J., Xie J. H., Shen M. Y., Nie S. P., Xie M. Y. (2018). Sulfated modification of polysaccharides: synthesis, characterization and bioactivities. *Trends in Food Science & Technology*.

[48] Yuan Y., Macquarrie D. (2015). Microwave assisted extraction of sulfated polysaccharides (fucoidan) from Ascophyllum nodosum and its antioxidant activity. *Carbohydrate Polymers*.

[49] Huang L. X., Huang M., Shen M. Y. (2019). Sulfated modification enhanced the antioxidant activity of Mesona chinensis Benth polysaccharide and its protective effect on cellular oxidative stress. *International Journal of Biological Macromolecules*.

[50] Hu H. B., Li H. M., Han M. H. (2020). Chemical modification and antioxidant activity of the polysaccharide from Acanthopanax leucorrhizus. *Carbohydrate Research*.

[51] Daudon M., Letavernier E., Frochot V., Haymann J. P., Bazin D., Jungers P. (2016). Respective influence of calcium and oxalate urine concentration on the formation of calcium oxalate monohydrate or dihydrate crystals. *Comptes Rendus Chimie*.

[52] Teodosio Melo K., Gomes Camara R., Queiroz M. F. (2013). Evaluation of sulfated polysaccharides from the brown seaweed Dictyopteris justii as antioxidant agents and as inhibitors of the formation of calcium oxalate crystals. *Molecules*.

[53] Zhang C. Y., Wu W. H., Wang J., Lan M. B. (2012). Antioxidant properties of polysaccharide from the brown seaweed sargassum graminifolium (Turn.), and its effects on calcium oxalate crystallization. *Marine Drugs*.

[54] Mosquera D. M. G., Ortega Y. H., Quero P. C., Martínez R. S., Pieters L. (2020). Antiurolithiatic activity of Boldoa purpurascens aqueous extract: an in vitro and in vivo study. *Journal of Ethnopharmacology*.

[55] Oliveira L. C. B. P., Queiroz M. F., Fidelis G. P. (2020). Antioxidant sulfated polysaccharide from edible red seaweed gracilaria birdiae is an inhibitor of calcium oxalate crystal formation. *Molecules*.

[56] Gomes D. L., Melo K. R. T., Queiroz M. F. (2019). In vitro studies reveal antiurolithic effect of antioxidant sulfated polysaccharides from the green seaweed caulerpa cupressoides var flabellata. *Marine Drugs*.

[57] Jin M. L., Wang Y. M., Huang M., Lu Z. Q., Wang Y. Z. (2014). Sulphation can enhance the antioxidant activity of polysaccharides produced by _Enterobacter cloacae_ Z0206. *Carbohydrate Polymers*.

[58] Wang Z. J., Xie J. H., Kan L. J. (2015). Sulfated polysaccharides from _Cyclocarya paliurus_ reduce H_2_O_2_-induced oxidative stress in RAW264.7 cells. *International Journal of Biological Macromolecules*.

[59] Wang J. M., Hu Y. L., Wang D. Y. (2010). Sulfated modification can enhance the immune-enhancing activity of lycium barbarum polysaccharides. *Cellular Immunology*.

